# Cost-effectiveness of Childbirth Strategies for Prevention of Mother-to-child Transmission of HIV Among Mothers Receiving Nevirapine in India

**DOI:** 10.4103/0970-0218.62550

**Published:** 2010-01

**Authors:** Kanchan Mukherjee

**Affiliations:** Centre for Health Policy, Planning and Management School of Health Systems Studies, Tata Institute of Social Sciences, Deonar, Mumbai - 400 088, Maharashtra, India

**Keywords:** Cost-effectiveness, decision analysis model, sensitivity analysis, prevention of mother-to-child transmission of HIV, vaginal delivery, caesarian delivery, nevirapine

## Abstract

**Background:**

Mother-to-child transmission of HIV is an important mode of spread of HIV in India. With strategies like caesarian section and nevirapine therapy, this spread has been reduced. However, they have costs attached. In this context, this paper attempts to compare the cost-effectiveness of alternative childbirth strategies among HIV-positive mothers receiving nevirapine.

**Materials and Methods:**

Using sentinel surveillance data from three districts in Tamil Nadu, a model was created to test the cost-effectiveness of vaginal delivery against elective caesarian section among mothers receiving nevirapine. Sensitivity analysis was applied to evaluate cost per HIV infection prevented.

**Results:**

Vaginal delivery is not only cheaper in HIV-infected mothers receiving nevirapine but also cost-effective as compared to elective caesarian section. The incremental cost for preventing an additional HIV infection through caesarian section was Rs. 76,000. Sensitivity analysis reveals that the findings are robust over a range of HIV transmission probabilities, 0.04-0.14 for vaginal delivery and 0.00-0.02 for caesarian section.

**Conclusions:**

From a clinical perspective, the findings suggest that pregnant HIV-infected women receiving nevirapine should consider the benefits of a cheaper and safer vaginal delivery. From an economic perspective, the findings support the strategy of vaginal delivery in mothers receiving nevirapine.

## Introduction

For millions of children, AIDS has drastically altered the experience of growing up. In 2007, it was estimated that 2.1 million children under age 15 were living with HIV, and 15 million children had lost one or both parents to the virus. Millions more have experienced deepening poverty, school dropout, and discrimination as a result of the epidemic.(1) Perinatal transmission of HIV is the primary and most common way that children below the age of 15 years become infected with HIV worldwide,(2) with more than half of transmission probably occurring late in pregnancy or during labor and delivery.(3) Mother-to-child transmission of HIV varies according to geographical region, delivery method, and breastfeeding practices and is estimated to be 21-43% in less-developed countries.(4)

Mother-to-child transmission becomes more important where heterosexual intercourse is the predominant mode of transmission, where women of childbearing age form a significant proportion of the infected population, and where fertility is high. This pattern exists in many of the low-income and developing countries, including India. Recent estimates suggest that 3.8% of the total HIV infections in this country are among children less than 15 years of age. India has an estimated 220,000 children infected by HIV/AIDS. It is estimated that 55,000 to 60,000 children are born every year to mothers who are HIV positive.(5) Tamil Nadu has been one of the high-HIV-prevalence states in India but has shown progress in controlling the epidemic. The HIV prevalence in antenatal cases in this state is 0.25%.(6) Without treatment, the babies born to these mothers have an estimated 30% chance of becoming infected during the pregnancy, during labor, or through breastfeeding during the first 6 months.(5) However, if the HIV status of the woman were known during the pregnancy, it would be possible to achieve reduction in transmission to 5-8% through strategies such as avoidance of breastfeeding;(7) administration of antiretroviral therapy (ART) during pregnancy, at delivery, and to new born;(8) and avoidance of invasive procedures during pregnancy and at delivery.(9,10) This has already been achieved in parts of Europe and the USA.(11,12)

The challenge for India is to determine how best to help meet the HIV targets of the Millennium Development Goals (MDG) and, more specifically, the target set by the United Nations General Assembly Special Session (UNGASS) on HIV/AIDS, which seeks to reduce the proportion of infants infected with HIV by 20%. There are several interventions—such as family planning, obstetric care, integrated counseling and testing centers (ICTC), antiretroviral (ARV) drugs, and artificial feeds—that can reduce the risk of transmission from an HIV-infected mother to her child. Though some of them are becoming more affordable, implementing these interventions poses complex challenges for health systems, communities, and individuals, especially in resource-poor settings. The focus of this study is on the benefits of adopting appropriate childbirth strategies along with the use of antiretroviral drug therapy.

Research has shown a 50% reduction in mother-to-child transmission if elective caesarian section (ECS) is carried out before labor and before membrane rupture(13) among HIV-infected women not taking ART or taking only zidovudine.(14) However, the available data regarding the benefit of caesarian section as an intervention to prevent transmission are largely from studies conducted in developed countries and/or before the widespread utilization of highly active antiretroviral therapy (HAART) for HIV-positive women. The risk of mother-to-child transmission of HIV according to mode of childbirth among HIV-infected women receiving HAART is unclear in the context of developing countries.

Preliminary data from developed countries suggest that ECS can benefit HIV-infected women with plasma viral loads <1000 copies/ml(15) or those receiving HAART.(16) The benefit of ECS for prevention of mother-to-child transmission of HIV may persist among women with low plasma viral loads because of compartmentalization of HIV reservoirs, i.e., a low plasma viral load does not necessarily indicate a low viral load in genital tract secretions. Therefore, an important issue to be addressed is assessment of the effectiveness of caesarian section for prevention of mother-to-child transmission of HIV among HIV-infected women receiving HAART.

While the national guidelines of prevention of mother-to-child transmission of HIV in India do recommend ECS for mothers infected with HIV, these guidelines were developed before HAART (such as nevirapine) became available for treatment of pregnant mothers. Also, in resource-poor settings such as India, the cost of ECS is often borne by the government and the amount can be significant. In this context, this paper attempts to compare the cost-effectiveness of alternative childbirth strategies among HIV-positive mothers receiving nevirapine in government ICTCs in Tamil Nadu, India. The findings of this study would be relevant for clinical case management as well as for formulation of state/national HIV/AIDS policy.

## Materials and Methods

The present study was designed as a retrospective cohort analysis with a comparison group. The study used the sentinel surveillance data from Tamil Nadu State AIDS Control Society (TANSACS). Sentinel surveillance data of mothers attending 26 government ICTCs in three districts (Chennai, Theni, and Dharmapuri) of the state during the period 2001-2005 were used for this study. These sites were selected as they provided nevirapine to mothers who tested HIV positive. Data meeting the following inclusion criteria were selected for analysis: HIV positivity of mothers, acceptance of the nevirapine regimen by mothers, availability of data on mode of childbirth, availability of data on 1 month postdelivery HIV status of child. This data was obtained with permission from TANSACS, which was implementing the prevention of mother-to-child transmission programme at these sites. Three hundred and sixty-two women fulfilled the inclusion criteria for this study.

**Figure 1:**
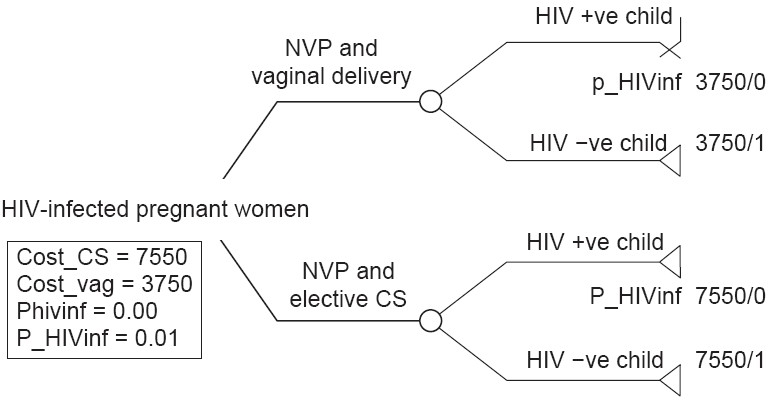
Decision analysis model

A decision analysis model from the clinical perspective was constructed [Figure 1]. The model compared two childbirth delivery strategies among mothers fulfilling the inclusion criteria: (1) usual care, in which HIV-infected mothers undergo vaginal delivery and (2) HIV-infected mothers are offered the option of elective caesarian section.

The model followed the data cohort of 362 HIV-infected women who received nevirapine from these centers to note their mode of childbirth and postdelivery HIV status of the child at 1 month. The model was developed with the probabilities of different childbirth strategies, their costs, and vertical transmission risk as witnessed in the cohort group. For the purpose of this study, only uncomplicated, noninstrumental vaginal delivery and uncomplicated elective caesarian delivery were considered. For this cohort, it is assumed that the HIV-infected women who received nevirapine therapy were in prenatal care by 36 weeks and did not breastfeed.

Cost data was provided by TANSACS. The cost of uncomplicated vaginal delivery plus nevirapine was Rs. 3,750 per mother, while the cost of uncomplicated caesarian section delivery plus nevirapine was Rs. 7,550 per mother. This included the cost of the drug, number of women given the drug, cost of personnel involved, hospitalization cost, and the direct and indirect costs related to mode of delivery (vaginal and caesarian). Costs of transport, productivity loss for mother, and other opportunity costs were not considered in the model.

Effectiveness was measured in terms of perinatal HIV transmissions prevented. The outcome measure was the number of cases of HIV among children tested at 1 month after birth. The outcomes of the decision analysis model were measured in terms of proportion of HIV infection prevented among the newborn. Effectiveness was measured by giving a weight of ‘1’ for every HIV-negative child and a weight of ‘0’ for every HIV-positive child.

The cost-effectiveness of the alternative childbirth strategies is evaluated in terms of their vertical transmission probabilities and the average costs per woman for each strategy. The costs, outcomes, and probabilities were entered into Tree-Age software for cost-effectiveness and sensitivity analysis.

One-way sensitivity analysis was undertaken to illustrate the impact of a range of HIV transmission probabilities for alternative childbirth strategies. Variables were deemed sensitive if their values were within the ranges found in literature. This was done based on a range of probabilities reported in literature of HIV transmission rate in vaginal and caesarian section deliveries. The range selected was from 0.04-0.14(17–19) for vaginal delivery and 0.00-0.02(14,18,20–22) for caesarian section.

## Results

**Figure 2:**
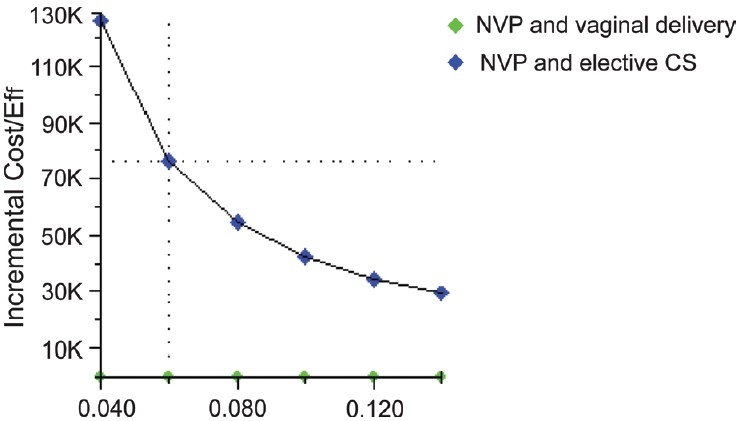
Sensitivity analysis with different probability of HIV transmission with vaginal delivery

**Figure 3:**
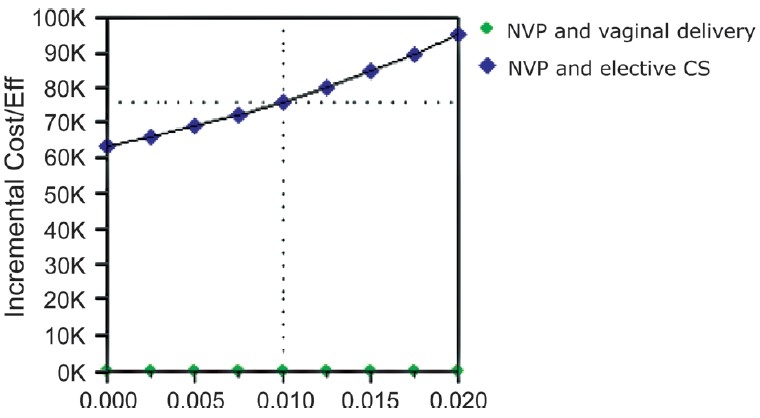
Sensitivity analysis with different probability of HIV transmission with caesarian delivery

Of the 362 mothers fulfilling the inclusion criteria, 295 had uncomplicated vaginal deliveries and 64 underwent uncomplicated elective caesarian section. Three had assisted breech delivery and were not considered for analysis. HIV transmission rate to child was 6.10% for vaginal delivery and 1.5% for ECS. On roll back the model suggests that vaginal delivery plus nevirapine is a cost-effective strategy as compared to caesarian section plus nevirapine. The incremental cost for preventing an additional HIV infection through caesarian section was Rs. 76,000. Sensitivity analysis results across the range of HIV transmission probabilities for vaginal deliveries are depicted in Figure 2 and for caesarian delivery in Figure 3. Text report of the same are presented in Tables 1 and 2.

**Table 1:** HIV transmission probabilities with vaginal delivery

P_HIVinf	Strategy	Cost	Incr cost	Eff	Incr Eff	C/E	Incr C/E (ICER)
0.04	NVP and vaginal delivery	3750.00	3800.00	0.96	0.33	3906.25	126666.67
	NVP and elective CS	7550.00		0.99		7626.26	
0.06	NVP and vaginal delivery	3750.00	3800.00	0.94	0.05	3989.36	76000.00
	NVP and elective CS	7550.00		0.99		7626.26	
0.08	NVP and vaginal delivery	3750.00	3800.00	0.92	0.07	4076.09	54285.71
	NVP and elective CS	7550.00		0.99		7626.26	
0.1	NVP and vaginal delivery	3750.00	3800.00	0.90	0.09	4166.67	42222.22
	NVP and elective CS	7550.00		0.99		7626.26	
0.12	NVP and vaginal delivery	3750.00	3800.00	0.88	0.11	4261.36	34545.45
	NVP and elective CS	7550.00		0.99		7626.26	
0.14	NVP and vaginal delivery	3750.00	3800.00	0.86	0.13	4360.47	29230.77
	NVP and elective CS	7550.00		0.99		7626.26	

P_HIVinf: Probability of HIV infection C/E: Cost/effectiveness; ICER: Incremental cost effectiveness ratio; NVP: Nevirapine; CS: Caesarian section

**Table 2:** HIV transmission probabilities with caesarian delivery

P_HIVinf	Strategy	Cost	Incr cost	Eff	Incr Eff	C/E	Incr C/E (ICER)
0	NVP and vaginal delivery	3750.00	3800.00	0.94	0.06	3989.36	6333.33
	NVP and elective CS	7550.00		1.00		7550.00	
0.0025	NVP and vaginal delivery CS	3750.00	3800.00	0.94	0.06	3989.36	66086.96
	NVP and elective CS	7550.00		1.00		7568.92	
0.005	NVP and vaginal delivery	3750.00	3800.00	0.94	0.06	3989.36	69090.91
	NVP and elective CS	7550.00		1.00		7587.94	
0.0075	NVP and vaginal delivery	3750.00	3800.00	0.94	0.05	3989.36	72380.95
	NVP and elective CS	7550.00		0.99		7607.05	
0.01	NVP and vaginal delivery	3750.00	3800.00	0.94	0.05	3989.36	76000.00
	NVP and elective CS	7550.00		0.99		7626.26	
0.0125	NVP and vaginal delivery	3750.00	3800.00	0.94	0.05	3989.36	80000.00
	NVP and elective CS	7550.00		0.99		7645.57	
0.015	NVP and vaginal delivery	3750.00	3800.00	0.94	0.05	3989.36	84444.44
	NVP and elective CS	7550.00		0.98		7664.97	
0.0175	NVP and vaginal delivery	3750.00	3800.00	0.94	0.04	3989.36	89411.76
	NVP and elective CS	7550.00		0.98		7684.48	
0.02	NVP and vaginal delivery	3750.00	3800.00	0.94	0.04	3989.36	95000.00
	NVP and elective CS	7550.00		0.98		7704.08	

P_HIVinf: Probability of HIV infection; C/E: Cost/Effectiveness; ICER: Incremental cost effectiveness ratio; NVP: Nevirapine; CS: Caesarian section

## Discussion

The results of the study indicate that among HIV-positive mothers receiving nevirapine, vaginal delivery is more cost-effective than ECS. This is true for a range of probabilities of HIV transmission rate. Prospective cohort studies have shown a decreased likelihood of perinatal HIV transmission with elective caesarian delivery(23,24) and an additive protective effect with zidovudine therapy.(17,18,20,21) Data from North America, Thailand, and Europe(15,25,26) suggest a benefit from caesarian section in HIV-infected pregnant women, even those with low viral loads. However, the apparent benefit of caesarian delivery needs to be weighed against its cost, especially in resource-poor settings. In resource-poor settings like India, there are additional complications such as lack of adequate infrastructure and skilled manpower. In this context, the findings of this study assume greater significance.

The role of mode of childbirth in the management of HIV-infected women must be assessed in light of the risks as well as benefits. HIV-infected pregnant women receiving nevirapine must be provided with all available information so that they can make informed decisions regarding the mode of childbirth to prevent transmission of infection to their children. With the availability of safe childbirth practices, individual women may consider the benefit of a cheaper vaginal delivery to outweigh the potential disadvantages of an ECS.

The findings from this study, from a cost perspective, seem to favor vaginal delivery among mothers taking nevirapine rather than caesarian section. The relatively low prices of antiretroviral drugs in India have made it possible to consider, for the first time, the financing of nevirapine therapy for pregnant women. While availability of skilled manpower and materials for elective caesarian section still remains an issue, the findings of this study suggest that safe vaginal delivery may be an acceptable solution for prevention of mother-to-child transmission of HIV in mothers on nevirapine. However, cost may not be the only determining factor in selecting the appropriate strategy for child delivery.

Since the outcome restricts itself to the 1-month HIV status of child, it is possible that some HIV infections in children may have been missed in this early period. Also, the effect of breastfeeding practice on outcome is not considered in this model. The costs considered do not take into account opportunity costs for mother, HIV diagnostic costs, and the costs of lifetime care of an HIV-infected child. The sample size is too small to allow generalization of the findings and hence policy implications are to be drawn with caution. As the study was based on secondary data, it was not possible to assess the reasons for which the mothers underwent vaginal delivery and the ethical implications, or whether counseling was conducted with respect to the protective effect of caesarian delivery.

## Conclusions

The economic factor is one of many factors influencing policy change. Although this study does provide critical economic evidence in favor of a change, factors outside the economic arena also influence policy formulation and change. This study had a small sample size and therefore more studies need to be done in different states of India to test the generalizability of the findings.
